# Metacognition and Perspective-Taking in Alzheimer’s Disease: A Mini-Review

**DOI:** 10.3389/fpsyg.2016.01812

**Published:** 2016-11-17

**Authors:** Elodie Bertrand, Jesus Landeira-Fernandez, Daniel C. Mograbi

**Affiliations:** ^1^Department of Psychology, Pontifícia Universidade Católica do Rio de JaneiroRio de Janeiro, Brazil; ^2^Institute of Psychiatry, Psychology and Neuroscience, King’s College LondonLondon, UK

**Keywords:** Metacognition, anosognosia, perspective-taking, dementia, Alzheimer’s Disease

## Abstract

Metacognition refers to the monitoring and regulation of cognitive processes and its impairment can lead to a lack of self-awareness of deficits, or anosognosia. In the context of different neurological and psychiatric disorders (e.g., traumatic brain injury, dementia, and schizophrenia), studies have shown that patients who present impairments in metacognitive abilities may be able to recognize such difficulties in others and in themselves when exposed to material in a third-person perspective. Considering that metacognitive impairments are an important characteristic of dementia, especially in Alzheimer’s Disease (AD), studies of the relationship between metacognition and perspective-taking may be relevant to improve the quality of life of people with dementia. The current paper first briefly addresses the theme of metacognition and the impact of metacognitive deficits in people with AD. The focus then turns to the relationship between metacognition and perspective-taking in different neurological and psychiatric disorders, particularly AD. This relationship is also discussed based on theoretical models, particularly the Cognitive Awareness Model (CAM). Specifically, the CAM suggests the existence of distinct memory systems for self- and other-information, an idea which is supported by neuroimaging findings. We suggest that the Default Mode Network, as it has been shown to be implicated in self vs. other processing and is affected early in AD, could explain the impact of perspective-taking on awareness of deficits in AD. Finally, we present possible clinical implications of the relationship between metacognition and perspective-taking in AD. Indeed, we considered the possibility of improving patient’s awareness through the use of a third-person perspective, which, consequently, may decrease the negative impacts of anosognosia in AD.

## Introduction

Increases in life expectancy are now well documented worldwide. With an aging population, the number of people who suffer from dementia is also increasing. According to Alzheimer’s Disease International (2015), 46.8 million people are currently living with dementia, and studies suggest that this number will increase in the near future, with estimations of double the number of people who are affected by dementia by 2030 (Wimo et al., 2006).

With the aim of promoting a better quality of life in people with dementia (PwD), the theme of metacognition is particularly relevant. The awareness of deficits relies on metacognitive abilities, which are shown to be compromised in PwD (Cosentino and Stern, 2005). In fact, a lack of awareness about behavioral and/or cognitive deficits, referred to as anosognosia, is a common characteristic of dementia (Morris and Hannesdottir, 2004; Mograbi et al., 2012), with an important impact on patients and their caregivers (Bertrand et al., 2013).

Within this context, this review first addresses the theme of metacognition and the impact of metacognitive deficits in PwD, especially in Alzheimer’s Disease (AD). The focus then turns to the relationship between metacognition and perspective-taking in AD. Studies that explore metacognitive abilities in different neurological and psychiatric disorders have suggested that the perspective through which information is presented may impact the patient’s awareness of his own deficits. Such studies have investigated the impact of perspective-taking on metacognitive abilities from two angles: self-observation through a third-person perspective (e.g., observing oneself from the outside through a video) and the evaluation of another person’s performance. The concept of perspective-taking is usually defined as the ability to take another person’s point of view (Ryskin et al., 2015). However, as it has already been done in the literature (Brugger, 2002; Besharati et al., 2015), in the current review, we will use the term “perspective-taking” to refer to the two perspectives explained above (self-observation in a third-person perspective and other-observation). The relationship between perspective-taking and awareness is discussed based on the Cognitive Awareness Model (CAM) (Agnew and Morris, 1998; Morris and Hannesdottir, 2004; Hannesdottir and Morris, 2007; Morris and Mograbi, 2013). Finally, we present possible clinical implications of this relationship in dementia.

## Metacognition

Metacognition is defined as the knowledge and reflective capacities one has concerning one’s own cognitive functioning (Flavell, 1979). Considering the original conceptualization proposed by Flavell (1979), metacognition can be divided into metacognitive knowledge (which represents acquired knowledge about one’s own and also other people’s cognitive processes) and metacognitive experience (which permits the updating of metacognitive knowledge and activation of strategies during cognitive processes). Nelson and Narens (1990) proposed another influential model of metacognition that comprises two levels of analysis, meta-level and object-level, which operate in an interrelated fashion. The meta-level receives information from the object-level through a monitoring process, and the meta-level regulates the object-level through a control process. There are some parallels between this model and the conceptualization of metacognition proposed by Flavell (1979). The monitoring process can be seen as “metacognitive knowledge,” and the control process can be seen as “metacognitive experience” in Flavell’s description of metacognition.

The literature on metacognitive abilities indicates that deficits in metacognition and anosognosia are referred to interchangeably. However, as highlighted by Souchay (2007), the two terms rely on different theoretical frameworks. The concept of anosognosia focuses on deficits in awareness in clinical populations, and the notion of metacognition is based on the functioning of awareness in normal populations. However, anosognosia and metacognition are closely related concepts. Based on the metacognitive theories described above, anosognosia can be seen as a deficit at the level of metacognitive knowledge or monitoring processes. Indeed, anosognosia is defined as the diminished ability to recognize the presence or appreciate the severity of deficits (Babinski, 1914). In other terms, it refers to a lack of awareness about deficits or the condition and is described in cases of various neurological conditions, such as aphasia, hemiplegia, and dementia (Stuss, 1991; Stuss et al., 2005; Ries et al., 2007; Mograbi et al., 2009). For example, in the case of anosognosia for hemiplegia, patients would state that they are capable of moving their paralyzed limb despite contradictory evidence (Orfei et al., 2007).

Unawareness of cognitive impairments are common characteristics of AD (Morris and Hannesdottir, 2004; Morris and Mograbi, 2013). The estimates of prevalence have varied widely across studies, ranging from 23 to 81%, likely because of differences in criteria and assessment methods (Antoine et al., 2004; Mograbi et al., 2012; Sousa et al., 2015). Nevertheless, studies that explore anosognosia in PwD with large samples have consistently reported a prevalence above 30% (Starkstein et al., 2006, 2007). In a large epidemiological study (*n* = 897), Mograbi et al. (2012) showed that 78% of PwD are unaware of their memory impairment. The multidimensional aspect of awareness also contributes to variations in the reported prevalence in dementia. In fact, there is considerable variability in the presentation and severity of anosognosia, with unawareness ranging from slight minimization to complete denial of problems (Clare et al., 2005). Additionally, awareness can differ according to its object (e.g., memory deficits, changes in activities of daily living, and the diagnosis itself), and it is not uncommon for patients to acknowledge problems in one sphere but not in another (neuropsychological deficits vs. behavioral and psychological symptoms) (Kotler-Cope and Camp, 1995; Vasterling et al., 1995).

## Relationship Between Metacognition and Perspective-Taking

Studies that explored the impact of self-observation found improvements in awareness when patients were allowed to see themselves in a video. Through the presentation of a case-study, Fotopoulou et al. (2009) showed that video self-observation can be used as an efficient technique to improve the awareness of motor deficits after stroke. These findings were replicated in other settings. Besharati et al. (2015) described two case-studies of patients who suffered from anosognosia for hemiplegia. At the time of the first experimental session, one patient was in an acute stage of recovery (15 days post-stroke), and the other patient was in the chronic stage (89 days post-stroke). Both patients presented a reinstatement of motor awareness after seeing themselves in a video, suggesting that self-observation through a third-person perspective positively influences metacognitive abilities. Similar results have been observed in the context of psychosis. For example, David et al. (2012) explored the efficacy of video-observation as a strategy to improve psychotic patients’ awareness. One group viewed a video of themselves during an acute psychotic episode, and a second group viewed a video of an actor who played the role of someone with acute psychosis. The authors showed that both groups improved their awareness regarding psychotic symptoms after watching the video. These findings reinforced previous results that also highlighted the potential use of video self-observation as an efficient intervention to improve insight in psychotic patients (Davidoff et al., 1998; Vikram et al., 2008).

The relationship between metacognition and perspective-taking has also been explored by studying the capacity to perceive deficits in someone else. For example, Ramachandran and Rogers-Ramachandran (1996) showed that, in the context of anosognosia for hemiplegia, patients were able to acknowledge the paralysis of others, even when they were unaware of their own paralysis. Similarly, Garrett et al. (2011) demonstrated that schizophrenia patients who lacked awareness of their own illness were able to accurately categorize clinical vignettes as cases of medical illness, no illness, or psychiatric illness. Altogether, these findings used different methods to assess awareness to highlight the possible relationship between metacognitive processes and perspective-taking in psychiatric and neurologic diseases.

### Metacognition and Perspective-Taking in the Context of AD

In the context of AD, a few studies have explored the issue of perspective-taking with regard to metacognitive abilities by asking patients with AD to evaluate the performance of their relatives. The results indicated that AD patients were generally accurate in predicting their caregiver’s performance, despite difficulties in evaluating their own performance. These results were attributable to deficits in the patients’ ability to update their knowledge of their own cognitive functioning (i.e., a monitoring deficit in the metacognitive process that is restricted to the self) (McGlynn and Kaszniak, 1991; Kaszniak and Zak, 1996). However, in a review of the neuropsychological bases of metamemory, which is a subtype of metacognition looking specifically at the control and monitoring of memory performances, Kaszniak and Zak (1996) argued that such an interpretation of these findings may be problematic because cognitive abilities in the patients’ relatives did not decline as sharply. Therefore, the patients may be able to make accurate evaluations of their caregivers’ performance even when relying on out-of-date knowledge of their relatives’ cognitive capacities. To resolve this issue, Duke et al. (2002) used a combination of two methods to assess awareness: predictions and post-dictions regarding performance and discrepancies between patient and informant reports. AD patients and their spouses were asked to estimate each other’s performance and the performance of a fictional patient who suffered from moderate cognitive impairment. The estimations were made before and after performing a verbal memory task and verbal fluency task. Before making the predictions, the participants were given normative data based on their own age range, their spouse’s age range, and the fictional patient’s age range. The results showed that AD patients overestimated themselves but made accurate predictions and post-dictions of their spouse’s performance. With regard to evaluating the fictional patient, both AD patients and their caregivers overestimated performance. These results, however, did not allow the determination of whether AD patients’ difficulties in awareness were self-specific or global. However, by comparing the predictions and post-dictions, the authors suggested that some monitoring abilities, in an on-line fashion, might be preserved in AD patients. Indeed, the results showed that they reduced the extent of overestimating their abilities after completing the task. To date, this is the only study that has compared the ability of AD patients to evaluate the performance of a well-known person (e.g., spouse) and an unknown person (e.g., fictional patient).

Using a vignette technique as an indirect method to assess awareness, Clare et al. (2012a) explored the ability of PwD (including AD, vascular dementia and mixed Alzheimer’s and vascular dementia) to appreciate the deficits of a fictional person. PwD, their caregivers, and older adult controls were asked to identify and offer advice for the problems that were described in the vignettes. Vignettes were created and described a short scenario of a person who presented a typical case of advanced or early stage dementia or a healthy older adult. The results showed that, when presented with these vignettes, the participants were able to correctly identify and offer appropriate advice for the problem that was described in the vignette. Additionally, some of the participants in the dementia group, even some who presented a limited level of awareness, identified themselves spontaneously with the situation that was presented in the vignette. These results led the authors to suggest that the vignette method (i.e., a third-person perspective method) could be useful to help PwD acknowledge their own deficits.

Based on the evaluation of a third person, Mograbi et al. (2014) investigated the attribution of difficulty for the self and for others. Patients with AD were asked to judge how difficult a task was for them and would be for someone else their own age. Two experiments were conducted using computerized tasks. Experiment 1 used reaction time tasks, and Experiment 2 used memory tasks. The level of task difficulty was controlled, leading to one easy and one difficult task in each experiment. In both experiments, the authors showed that AD patients predicted that others would perform as well as themselves, despite their cognitive deficits. This study also explored correlations with premorbid personality and showed that higher neuroticism and agreeableness were associated with the attribution of more difficulty for the self and less difficulty for others. These findings were interpreted by the authors as the poor ability of people with AD to put themselves in someone else’s position. The authors explained that the different findings between their results and Clare et al. (2012a) were attributable to methodological differences. Indeed, in Mograbi et al. (2014), the cognitive demand of the task, where participants had to imagine themselves as someone else, was higher than in Clare et al. (2012a), in which patients did have access to written vignettes. Altogether, these results suggest that patients with AD are more accurate when evaluating the performance of someone else (e.g., caregiver or fictional person) than they are for themselves. Nevertheless, the methods that were employed in these studies had distinctly different features (e.g., known vs. unknown person, vignettes vs. actual observation, prediction of performance vs. judgment of difficulty level), thus limiting synthesis of the findings. Moreover, to date, no study has explored the impact of self-evaluation through a third-person perspective, using for example video self-observation, on awareness level in AD patients. Further research is needed to better understand the impact of perspective-taking on the awareness of deficits in dementia. Additionally, possible symptoms, such as a deficit in familiarity recognition for self and other faces, should also be considered in studies investigating the impact of perspective-taking in dementia (Van den Stock et al., 2012, 2013). Finally, it is important to acknowledge the differences in the underlying mechanisms of metacognitive deficits in different types of dementia, such as AD and FTD (Salmon et al., 2008; Rosen, 2011) and, therefore, to take those into account when exploring self vs. other-information processing in dementia.

### Theoretical Explanation

**FIGURE 1:**
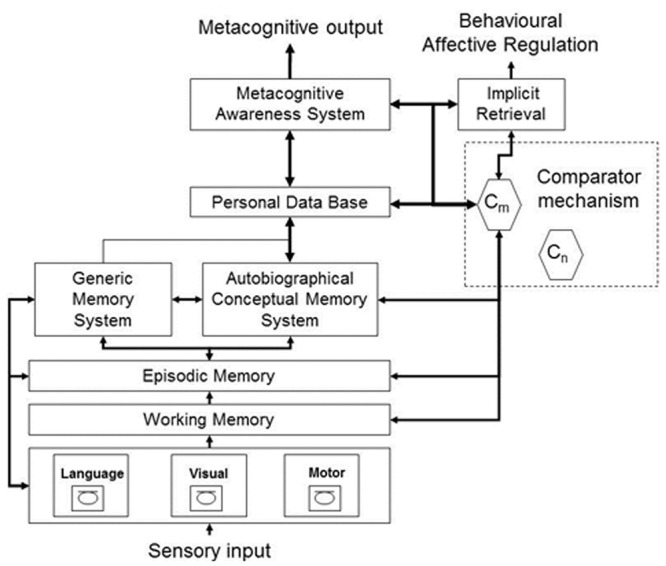
**Cognitive Awareness Model (CAM) Morris and Mograbi (2013)**.

As suggested by Morris and Mograbi (2013), the behavioral evidence may reflect the existence of different networks that are involved in self/other appraisal. The authors included this notion in a reformulated version of the CAM (Agnew and Morris, 1998; Morris and Hannesdottir, 2004; Hannesdottir and Morris, 2007; Morris and Mograbi, 2013), which is a neurocognitive model of anosognosia, accounting for the heterogeneous presentation of disordered awareness, through domain-specific modules (see **Figure 1**). In fact, they proposed different memory records for self- and other-information. First, the Personal Database, which contains semanticized representations about the self, and the Autobiographical Conceptual Memory System, which contains lifetime knowledge concerning experienced events, allow the appraisal of self-evaluation. Both are relying on personal semantics, which is acquired through personal, social, and cultural experiences. Second, these memory systems are separated from the Generic Memory System, which stores other material that permits evaluations of others and is based on general semantic knowledge.

### Neuroimaging Evidence

Recent neuroimaging evidence supports the idea of different neural bases for processing self vs. non-self information. For example, Ruby and Decety (2001, 2003, 2004) explored the neuroanatomical correlates of perspective-taking regarding different domains: motor, conceptual, and emotional. The authors showed that, for these three domains, the frontopolar, somatosensory, and inferior parietal cortices are implicated in the process of self vs. other distinction. As underscored by Ruby et al. (2009), some of these brain areas, such as the orbitofrontal cortex and temporoparietal junction, have also been shown to be related to measures of anosognosia in AD. Additionally, these brain regions are partially overlapping with those that comprise the default mode network (DMN). Indeed, the DMN, activated when participants are not engaged in a specific cognitive task, includes the frontal, parietal and temporal regions (for review see Buckner et al., 2008). Moreover, this network has been shown to be affected early by AD neurodegenerative process (for review see Mevel et al., 2011) and, as suggested by Bond et al. (2016), the impairment of the DMN by AD neurodegeneration could explain the differences in metacognitive abilities depending on the perspective through which the information is presented. However, to date, no neuroimaging study has specifically explored the issue of perspective-taking (first- vs. third-person perspectives) regarding metacognitive processes in AD.

### Clinical Implications

The findings regarding the relationship between metacognition and perspective-taking may have important clinical implications. In fact, studies have shown that the lack of awareness is related to higher engagement in risk situations, earlier institutionalization, more difficulties with treatment adherence, and greater caregiver burden, all of which increase the financial impact on society (Starkstein et al., 2007; Arlt et al., 2008; Bertrand et al., 2013; Horning et al., 2014). Interventions that increase awareness may eventually bring important clinical benefits. Throughout this review, we highlighted the possibility of using third-person perspective paradigms to do so. This type of intervention has already been successfully used with anosognosic patients with neurological or psychiatric disorders, such as hemiplegia after stroke or schizophrenia (Vikram et al., 2008; Fotopoulou et al., 2009; David et al., 2012; Besharati et al., 2015). However, when using interventions to improve awareness, caution needs to be taken with the patient’s emotional state. Studies have shown that awareness regarding deficits in AD may lead to negative mood changes, including depressive symptoms (Harwood et al., 2000; Clare et al., 2012b). Thus, further efforts need to be made by clinicians to acknowledge changes in their patients’ mood states during interventions that attempt to decrease the level of anosognosia.

## Conclusion

In summary, there is preliminary evidence suggesting that AD patients might show better awareness when evaluating abilities of others or themselves in a third-person perspective. These findings are supported by the CAM, which suggests distinct memory systems for self- and other-information, and also by neuroimaging studies, highlighting different neural bases for self and non-self processing. The early impairment of the DMN in AD could explain the relationship between metacognition and perspective-taking. Regarding possible clinical implications, we believe that improvements in patient’s awareness through the use of a third-person perspective may decrease the negative impacts of anosognosia in AD. However, practitioners, psychologists, and other health professionals who are involved in patient’s rehabilitation must be aware of the potential psychological and emotional consequences of increasing patients’ awareness of their condition and impairments.

## Author Contributions

EB collected the materials and resources needed for this review. EB wrote this paper, which is derived from EB’s doctoral studies for which DM and JL-F are co-supervisors. DM and JL-F provided suggestions on the structure of the paper and revised each draft.

## Conflict of Interest Statement

The authors declare that the research was conducted in the absence of any commercial or financial relationships that could be construed as a potential conflict of interest.
